# Targeting repair of the vascular endothelium and glycocalyx after traumatic injury with plasma and platelet resuscitation

**DOI:** 10.1016/j.mbplus.2022.100107

**Published:** 2022-03-17

**Authors:** Mark Barry, Shibani Pati

**Affiliations:** a University of California, San Francisco, Department of Surgery. 513 Parnassus Ave., San Francisco, CA 94143, United States; b University of California, San Francisco, Department of Laboratory Medicine. 513 Parnassus Ave., San Francisco, CA 94143, United States

**Keywords:** Endothelial glycocalyx, Hemorrhagic shock, Trauma, Vascular permeability, Plasma, Platelets, EOT, Endotheliopathy of trauma, FFP, Fresh frozen plasma, TIC, Trauma-induced coagulopathy

## Abstract

Severely injured patients with hemorrhagic shock can develop endothelial dysfunction, systemic inflammation, and coagulation disturbances collectively known as the endotheliopathy of trauma (EOT). Shedding of the endothelial glycocalyx occurs early after injury, contributes to breakdown of the vascular barrier, and plays a critical role in the pathogenesis of multiple organ dysfunction, leading to poor outcomes in trauma patients. In this review we discuss (i) the pathophysiology of endothelial glycocalyx and vascular barrier breakdown following hemorrhagic shock and trauma, and (ii) the role of plasma and platelet transfusion in maintaining the glycocalyx and vascular endothelial integrity.

## Highlights

• Endothelial glycocalyx shedding is a key instigator of the endotheliopathy of trauma.
• Plasma and platelet transfusions preserve vascular integrity in pre-clinical models.
• However, platelets may be less effective than plasma in preserving the glycocalyx.

## Introduction

Traumatic injury is an important cause of death globally and the leading cause of death in the United States among individuals aged 1–44 [1], [2]. Hemorrhage accounts for approximately 30–40% of trauma-related deaths [3], and patients who survive hemorrhagic shock face significant morbidity as well as increased long-term mortality [4]. One of the major principles in the management of patients with hemorrhagic shock due to trauma is damage control resuscitation, which includes using balanced resuscitation with a 1:1:1 ratio of red blood cells, fresh frozen plasma (FFP), and platelets, and minimizing crystalloid infusion [5], [6]. Damage control resuscitation allows for rapid expansion of intravascular volume, restoration of oxygen-carrying capacity, and optimization of hemostasis [7]..

The benefits of resuscitation with plasma and platelets also likely extend further to include protection of the vascular endothelium after hemorrhagic shock. Hemorrhagic shock is known to cause shedding of the endothelial glycocalyx and damage to the vascular endothelium systemically. This is a key element of the endotheliopathy of trauma (EOT), which is characterized by endothelial barrier compromise, dysfunctional coagulation, and inflammation, ultimately contributing to multiple organ failure [8], [9], [10], [11]. In this review we discuss the pathophysiology of hemorrhagic shock induced endothelial damage and the role that plasma and platelet products have in targeting the endothelial glycocalyx and protecting vascular endothelial barrier integrity after injury.

## Composition and physiology of the endothelial glycocalyx

The glycocalyx refers to the pericellular matrix attached to the cell membrane of multiple cell types. The endothelial glycocalyx is comprised of a carbohydrate-rich network of proteoglycans, glycoproteins, and glycosaminoglycans that line the inner surface of the vascular endothelium. These major components of the endothelial glycocalyx include membrane-bound proteins such as syndecans and glypicans, cell surface receptors such as selectins and integrins, and negatively charged polysaccharides such as heparan sulfate, chondroitin sulfate, and hyaluronic acid [12], [13]. This network of molecules interacts with soluble plasma- and endothelium-derived components forming a dynamic and physiologically active surface layer between endothelial cells and the circulating blood [14], [15], [16].

The endothelial glycocalyx has a vasculoprotective role which is essential to normal vascular barrier function, serving a number of important physiologic functions, including regulation of vascular permeability, coagulation, leukocyte-endothelial and platelet-endothelial interactions, microvascular rheology, and transduction of the mechanical forces of blood flow into cell signaling (i.e. mechanotransduction) [15], [17], [18], [19], [20], [21]. The endothelial glycocalyx, however, is fragile and can break down quickly in response to ischemia or inflammation, shedding its components and resulting in fluid extravasation, edema, leukocyte and platelet adhesion, hypercoagulability, loss of flow-responsive vasodilatation, and impaired microcirculation [22], [23], [24], [25], [26]. While the precise mechanisms of glycocalyx disruption are not fully understood, reactive oxygen species and pro-inflammatory cytokines have been shown to lead to the release or increased activity of enzymes called sheddases, which cleave components of the glycocalyx from the endothelial cell surface [27], [28]. Key sheddases that have been identified include matrix metalloproteinases (MMPs) and A Disintegrin and Metalloproteinases (ADAMs), which cleave syndecan ectodomains, as well as heparanase and hyaluronidases, which cleave heparan sulfate moieties and hyaluronic acid, respectively [27], [29], [30], [31], [32], [33].

## The pathophysiology of the endothelial glycocalyx and vascular endothelium in hemorrhagic shock and trauma

One of the first insults to the vasculature in hemorrhagic shock and trauma is compromise of the endothelial glycocalyx, which can occur within minutes after injury, leading to loss of vascular barrier integrity and dysregulation of the important physiologic functions described above [17], [21]. Disruption of the endothelial glycocalyx is thought to be a central instigating point for the EOT, in which the downstream effects of hemorrhagic shock and trauma lead to endothelial dysfunction, coagulopathy, edema, and organ dysfunction, ultimately with poor outcomes.

**Fig. 1:**
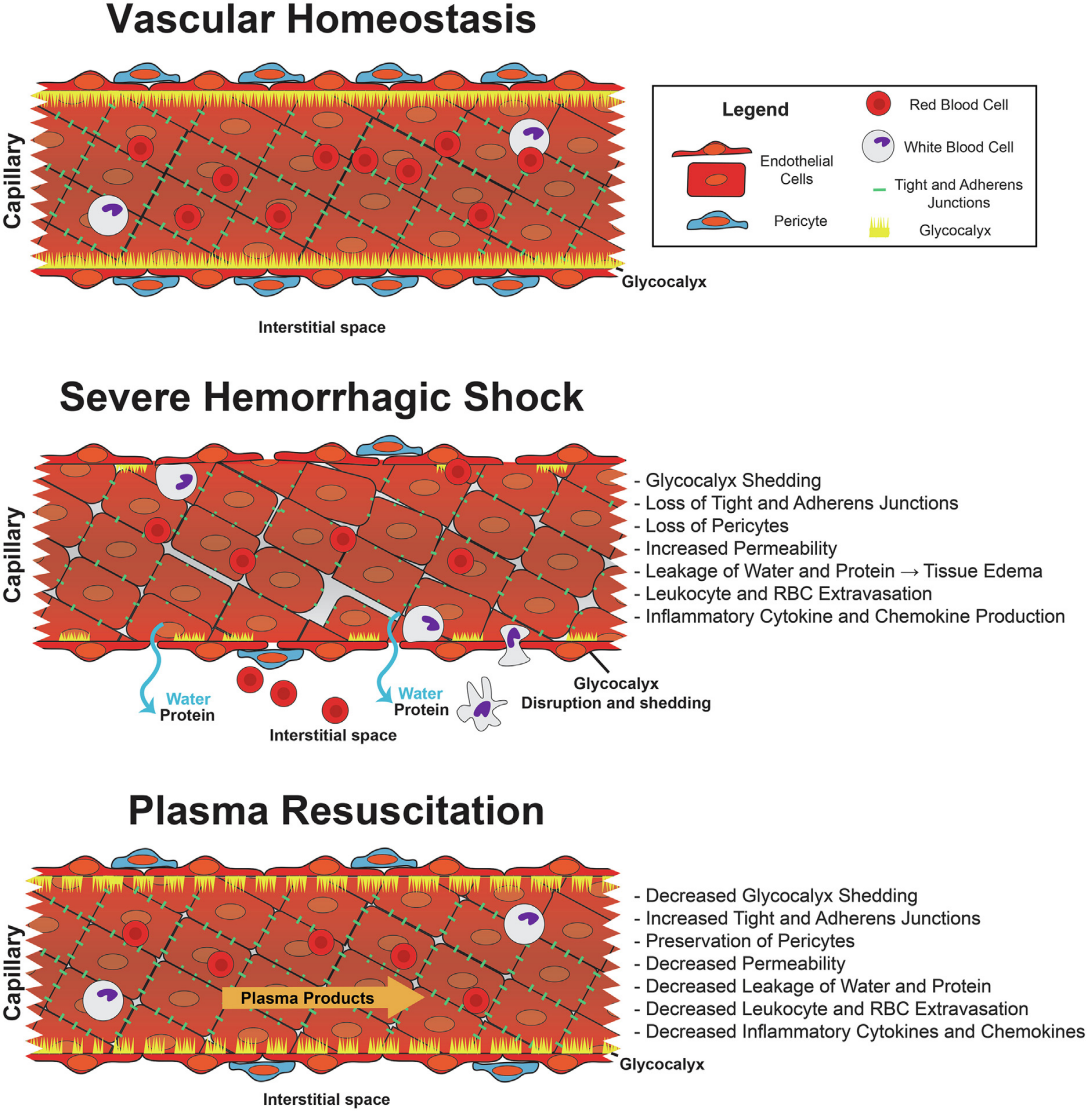
Hemorrhagic Shock and Trauma Induce Endothelial Glycocalyx Shedding and Vascular Permeability. This figure depicts longitudinal cross sections of a blood vessel. The top image corresponds to the resting endothelium with the endothelial glycocalyx fully intact. After hemorrhagic shock and trauma, the vasculoprotective endothelial glycocalyx is shed, and tight junctions and adherens junctions are lost. Gaps form between endothelial cells that lead to extravasation of proteins, fluid, and inflammatory cells. Resuscitation with plasma products restores and preserves the endothelial glycocalyx and vascular barrier function.

The endothelial glycocalyx as described above is a fragile matrix that is vulnerable to both acute and chronic stressors. Systemic inflammatory mediators, catecholamine release, hypoxia, acute hyperglycemia, and enzymes such as matrix metalloproteinases and heparanases released by damaged tissue and leukocytes are all thought to contribute to shedding of the glycocalyx components after hemorrhagic shock and trauma [23], [34], [35], [36], [37], [38]. Several of these shed components directly potentiate injury by acting as damage-associated molecular patterns (DAMPs) contributing to systemic inflammation or, in the case of heparan sulfates, by potentially inducing auto-heparinization which may contribute to coagulopathy [34], [39], [40]. Furthermore, loss of the protective endothelial glycocalyx barrier in addition to loss of endothelial tight junction proteins induced by hemorrhagic shock and trauma increases vascular permeability, leading to capillary leak (Fig. 1) [21], [41], [42], [43], [44].

At baseline in non-injured vessels, endothelial cells promote anticoagulant properties, prevent platelet activation and aggregation, and counteract inflammation [45]. After breakdown of the endothelial glycocalyx, however, exposure of the endothelial cell surface allows for the initiation of platelet- and leukocyte-adhesion and thrombus formation, which can be non-specific and in locations other than the injured area [46], [47]. In addition to glycocalyx shedding, hypoxia, proinflammatory cytokines such as TNF-α and IL-6, and circulating thrombin also contribute to endothelial cell activation and dysfunction, initiating a cascade of intracellular signaling resulting in further release of cytokines, chemokines, and growth factors involved in the innate immune response to shock and injury [21], [48], [49], [50], [51], [52]. Activated endothelial cells also release von Willebrand factor (VWF) and tissue plasminogen activator (t-PA), promoting platelet binding and activating fibrinolytic pathways, respectively [53], [54]. Altogether, loss of the endothelial glycocalyx and activation of endothelial cells result in aberrant clotting, inflammation, and vascular leak that can occur throughout the systemic endothelium. Studies in preclinical models have indeed demonstrated that hemorrhagic shock breaks down the endothelial glycocalyx, disrupts endothelial tight and adherens junctions, and results in organ edema, inflammation, thrombus formation, inflammatory cell activation, and potential organ failure [20], [33], [34].

While shedding of the endothelial glycocalyx is thought to affect the systemic vascular endothelium as the glycocalyx is ubiquitous, there may be organ-based differences. Recently Abdullah et al. [55] evaluated glycocalyx shedding after hemorrhagic shock in a rat model by staining multiple organs with a syndecan-1 antibody. This demonstrated that the greatest glycocalyx disruption occurred in the vasculature of the lungs and intestine, where the highest levels of endothelial reactive oxygen species were also measured, and shedding to a lesser extent occurred in the brain, heart, and skeletal muscle. Interestingly, there was no change in glycocalyx thickness in liver vasculature and there was increased glycocalyx staining in the kidney, though the authors did speculate the possibility that this may have been affected by deposition of systemically shed glycocalyx components in the glomeruli. In other studies, shedding of the endothelial glycocalyx from renal capillaries has been observed after ischemia–reperfusion during organ transplantation [56] and in mouse models of ischemia–reperfusion injury or sepsis [57], [58]. Discrepancies between studies may also be related to different methods of glycocalyx assessment (e.g. intravital microscopy, electron microscopy, or immunostaining).

## Endothelial dysfunction and trauma-induced coagulopathy (TIC)

While the mechanistic relationship between trauma-induced coagulopathy (TIC) and the EOT is not fully understood, these pathophysiologies are likely intertwined [54], [59]. TIC refers to the development of coagulation abnormalities following traumatic injury which include a range of phenotypes from hypocoagulability (contributing to uncontrolled bleeding and shock) to hypercoagulability (resulting in thromboembolism and multi-organ failure). TIC is important clinically as it results in substantial mortality and, among survivors, both short and long-term morbidity [54].

Multiple factors influence the development of TIC, including endothelial activation, platelet dysfunction, and immune system activation, which have been recently reviewed extensively by Moore et al. [54] The endothelium exerts critical regulatory functions over the coagulation cascade, hemostasis and fibrinolysis [54], [59]. Examples of these regulatory functions include binding of antithrombin III, thrombomodulin expression, expression of endothelial protein C receptor (EPCR), and release of the tissue factor pathway inhibitor (TFPI) [47], [60]. At the level of platelet adhesion and activation, hemostatic control is maintained by production and release of vWF as well as cleavage of ultra-large vWF by ADAMTS13 [60], [61]. Endothelial cells also regulate fibrinolysis via the release of t-PA, induction of plasminogen activator inhibitor-1, and stimulation of urokinase-type plasminogen activator along with its receptor [62], [63].

While the endothelial surface layer at baseline has anticoagulant properties, the exact role that glycocalyx shedding and the disrupted endothelium play in the various phenotypes seen in TIC or in the transition between hypocoagulable and hypercoagulable states is unclear. There are multiple ways in which glycocalyx shedding and disrupted endothelium can contribute to both hypocoagulable and hypercoagulable states. When endothelial glycocalyx shedding occurs, for example, the disrupted endothelial surface becomes exposed, increasing platelet-vessel wall interactions leading to increased platelet adhesion, fibrin formation, and microvascular thrombosis [22], [64], [65]. Endothelial cell activation after trauma also contributes to a localized procoagulant milieu [59], [66]. At the same time, the shed components themselves may impact systemic coagulopathy. One group has shown that shed heparan sulfate domains may in fact induce auto-heparinization leading to inhibition of coagulation factor activity [40]. Shed glycocalyx components have also been shown *in vitro* to inhibit both platelet aggregation and fibrinolysis [67]. In summary, TIC is a complex process, the full details of which are beyond the scope of this review, however it is apparent that endothelial activation and shedding of the glycocalyx are critical components in the development of aberrant coagulation in hemorrhagic shock and trauma.

## Glycocalyx degradation and endotheliopathy in trauma patients

Shedding of the endothelial glycocalyx has been evaluated in clinical studies and is known to occur in trauma patients, correlating with poor outcomes [11], [21], [41], [42], [68], [69], [70]. Various plasma biomarkers have been used to evaluate endothelial glycocalyx shedding in trauma patients, most commonly syndecan-1, but also thrombomodulin, hyaluronic acid, heparan sulfate, and chondroitin sulfate, all of which are key components of the endothelial glycocalyx. All of these proteins have been found to be elevated in the circulation after trauma [21], [41], [70]. Both the overall burden of injury and severity of hemorrhagic shock are thought to contribute to the likelihood of developing and degree of the EOT [10]. Injury severity scores, lower blood pressure, and the need for transfusion have been shown to correlate with circulating syndecan-1 levels [68], [70]. Rates of EOT, based on elevated biomarker levels, are higher in patients with combined polytrauma and traumatic brain injury (TBI) compared to polytrauma patients, and isolated TBI patients have the lowest rates of EOT [71]. Glycocalyx shedding also appears to occur more commonly among patients with blunt compared to penetrating injury, which likely reflects an overall greater burden of tissue injury [68], [72]. Hemorrhagic shock has also been found to be a potent driver of endothelial glycocalyx component shedding among patients with polytrauma, resulting in higher plasma syndecan-1 levels and markers of acute organ injury [42]. Moreover, shedding of these glycocalyx components has been associated with an increased risk of sepsis and mortality even after adjusting for injury severity [68], [69], [70], [73].

While circulating levels of these proteins have been used as biomarkers of endothelial injury in multiple other diseases as well [74], [75], [76], [77], [78], one potential limitation in their ability to evaluate glycocalyx shedding is that they are not specific only to the endothelial glycocalyx. Syndecan-1, the most commonly used biomarker, is expressed on multiple cell types such as epithelial cells and leukocytes which are also damaged and activated in injury [79]. Analysis of the tissue-specific pattern of syndecan-1 gene expression demonstrates that syndecan-1 is expressed in substantially higher amounts in bronchial epithelial cells, for example, compared to endothelial cells [80]. The percent of circulating syndecan-1 originating from endothelial versus non-endothelial surfaces is not known. However, studies evaluating microcirculatory perfusion in patients after trauma or during cardiopulmonary bypass have found that these biomarkers, including syndecan-1, do closely correlate with impaired microcirculatory flow dynamics in sublingual mucosa, suggesting that these biomarkers likely do reflect glycocalyx shedding and endotheliopathy [81], [82]. Notably, there are no established methods otherwise to directly measure vascular integrity and the endothelial glycocalyx in patients. Measuring levels of multiple glycocalyx proteins in patient samples, as opposed to a single biomarker, may improve accurate evaluation of the integrity of the endothelial glycocalyx.

## Plasma protection of the endothelial glycocalyx and vascular endothelial integrity

The detriments of large volume crystalloid resuscitation, including dilutional coagulopathy and metabolic acidosis, have led to a paradigm shift in the management of patients with hemorrhagic shock. Now, early administration of balanced ratios of blood products is understood to be critical in the management of these patients to rapidly restore volume and oxygen-carrying capacity and optimize hemostasis [7], [8], [83], [84]. The decreased mortality resulting from balanced blood product ratios may be due in part to the ability of plasma-based resuscitation to prevent and mitigate endothelial cell injury. Numerous pre-clinical studies have demonstrated that plasma preserves the endothelial glycocalyx and vascular barrier integrity, thus mitigating the EOT [85]. (Fig. 1).

**Fig. 2:**
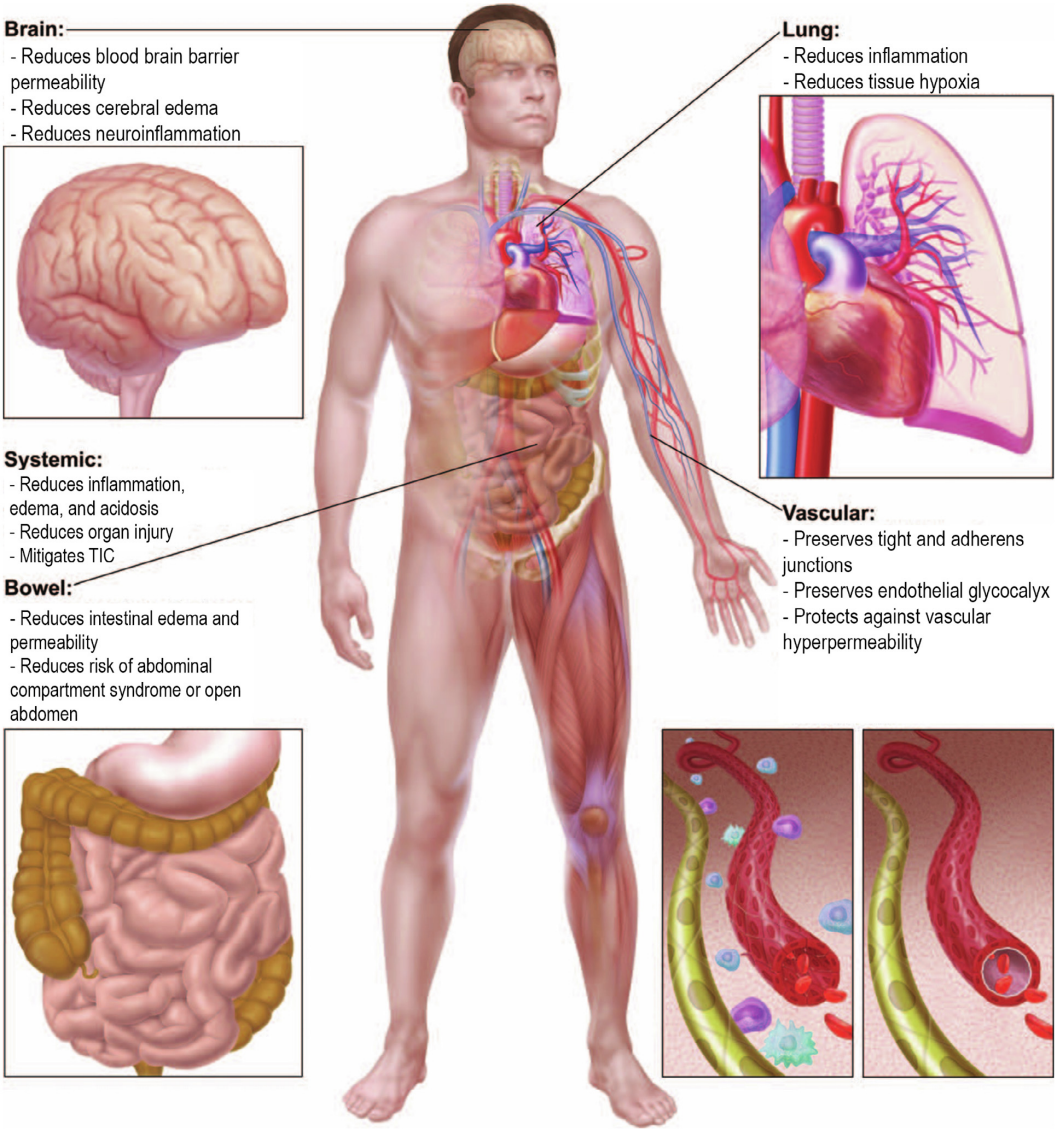
Early Plasma Transfusion Mitigates Organ-Specific and Systemic Dysfunction Following Trauma. Traumatic injury combined with hemorrhagic shock result in numerous systemic effects including a proinflammatory state, increased vascular permeability, endothelial glycocalyx shedding, coagulopathy, interstitial edema, and tissue hypoxia. Early plasma transfusion has been demonstrated to inhibit vascular permeability, mitigate trauma-induced coagulopathy (TIC), and decrease numerous organ-specific effects of injury. Image adapted from Watson, Pati, and Schreiber (2016) [158].

One of the first studies to investigate the effects of plasma on endothelial cells was conducted by Pati and colleagues in 2010, where it was discovered that addition of 10% plasma to the surface of leaky endothelial monolayers *in vitro* rapidly resulted in decreased endothelial permeability, decreased leukocyte adhesion, and restoration of endothelial adherens junctions, which are critical regulators of paracellular permeability [86], [87] Soon after, Kozar et al. (2011) investigated the effects of plasma on the endothelial glycocalyx *in vivo* and found that hemorrhagic shock in rats decreased endothelial glycocalyx thickness, which was restored in part by plasma resuscitation but not lactated Ringer’s (LR) solution. This was visualized by scanning electron microscopy (SEM). Similarly, they found that plasma resuscitation was associated with increased pulmonary cell surface syndecan-1 staining and decreased lung injury and edema [88]. Expanding on this work in a similar model of hemorrhagic shock and trauma in mice, Peng et al. (2013) demonstrated similar findings in terms of syndecan-1 shedding and pulmonary syndecan-1 expression, and also showed that this was associated with increased pulmonary vascular permeability and inflammation. Plasma resuscitation abrogated these effects, while LR did not [89]. Around the same time, using intravital microscopy of the cremaster muscle in rats subjected to hemorrhagic shock, others showed that rats resuscitated with LR or Hextend (an artificial colloidal solution) had a 50% decrease in endothelial glycocalyx thickness and increased circulating syndecan-1 levels compared to sham rats or rats resuscitated with plasma [90]. In addition to mitigating endothelial glycocalyx breakdown, plasma has protective effects on adherens junctions, which are intercellular junctions that control vascular permeability to plasma proteins [91], [92], [93]. Plasma but not LR preserves adherens junctions on endothelial cells challenged with vascular endothelial growth factor (VEGF) or thrombin as well as in the pulmonary vasculature in mice after hemorrhagic shock [43], [94], [95]. The globally stabilizing effects of plasma resuscitation on the vasculature and organ systems are depicted in Fig. 2.

Which specific factors within plasma that are responsible for its vasculoprotective effects remain elusive. Hundreds of biologically active proteins have been identified in FFP in addition to clotting factors [96]. Particular attention has been given to albumin and fibrinogen, which are discussed further below. Plasma also has abundant levels of adiponectin [97], sphingosine-1-phosphate (S1P) [98], [99], [100], and angiopoietin-1 (Ang-1) [101], [102], which are known regulators of paracellular permeability. It is likely that the combination of multiple factors and peptides in plasma contributes to its vasculoprotective effects in hemorrhagic shock. Interestingly, other products that are derived from plasma, such as four-factor prothrombin complex concentrate (which contains high concentrations of factors II, VII, IX, and X, and proteins C and S) and cryoprecipitate (which contains high concentrations of fibrinogen, von Willebrand factor, factor VIII, factor XIII, and fibronectin) likely contain many of these same proteins and have also been shown to mitigate the EOT and lung injury in mouse models of hemorrhagic shock and trauma [94], [103].

Albumin, the most abundant protein in plasma and the major contributor to plasma colloid oncotic pressure, has been shown to have immunomodulatory effects, scavenge free radicals, and physiologically bind to the endothelial glycocalyx and thereby contribute to the maintenance of normal vascular permeability [99], [104]. In a rat model of hemorrhagic shock, endothelial glycocalyx thickness dropped to 42% of baseline in rats resuscitated with normal saline (NS) or LR, whereas there was partial restoration with albumin (81% of baseline) and complete restoration of the glycocalyx with FFP resuscitation. Both albumin and FFP decreased circulating syndecan-1 levels compared to NS or LR and also normalized microvascular permeability similar to sham levels [105]. Moreover, resuscitation with albumin compared to NS has also been found to improve mesenteric microcirculation and reduce leukocyte rolling and adhesion in rats subjected to hemorrhagic shock [106]. However, in a mouse model of hemorrhagic shock, albumin administered at a concentration similar to what is found in four-factor prothrombin complex concentrate (4F-PCC) had only a mild inhibitory effect on pulmonary vascular permeability compared to 4F-PCC and FFP, which appeared more potent [103]. Altogether these studies suggest that albumin has therapeutic benefits compared to crystalloid but does not fully account for plasma’s ability to protect the vascular endothelium after hemorrhagic shock.

Fibrinogen, a clotting factor also abundant in plasma and the first factor to be depleted after massive haemorrhage [107], may also contribute to vascular endothelial protection. *In vitro* in pulmonary endothelial cell culture, fibrinogen was found to associate with and stabilize cell surface syndecan-1 and also had protective effects on barrier integrity. Furthermore, plasma that was deprived of fibrinogen lost its protective effects [108]. *In vivo* in a mouse model of hemorrhagic shock, cryoprecipitate, which is used to replete fibrinogen in massively bleeding patients, replicated many of the effects of FFP in mitigating pulmonary vascular permeability and inflammation in the lung [94]. Fibrinogen concentrate also preserved pulmonary syndecan-1 mRNA and decreased alveolar protein permeability after hemorrhagic shock in a similar model [109]. Proposed mechanisms for fibrinogen’s effects include shielding of syndecan-1 cleavage sites from sheddases and/or activation of an intra-cellular signaling pathway such as PAK1 which helps to preserve endothelial barrier integrity [110], [111].

One challenge with early plasma administration after traumatic injury involves the transportation, storage, and thawing of frozen blood products, especially in austere or remote environments [112], [113]. In the U.S., FFP may be stored at 4 °C for up to five days once thawed. However, between day 0 and day 5 after thawing, the coagulation factor levels, protective effects on endothelial permeability *in vitro*, and ability to restore mean arterial pressure in rats after hemorrhagic shock all decline [86], [114]. Dried plasma products, such as lyophilized plasma (LP) or spray-dried plasma (SDP), overcome these logistical burdens, can reduce time to plasma transfusion in injured patients [115], and have been shown in preclinical models to have vasculoprotective effects similar to FFP. Compared to FFP, LP demonstrated similar potent effects on decreasing endothelial cell monolayer permeability *in vitro* and reducing pulmonary vascular permeability, injury, and inflammation in mice subjected to hemorrhagic shock [95]. Spray-dried plasma also replicated the protective effects of FFP *in vitro* and *in vivo* on pulmonary vascular permeability and inflammation in this same mouse model [43], [116].

Never-frozen liquid plasma, which has a maximum potential refrigerated shelf life of 26–40 days depending on collection conditions, is another attractive alternative to FFP as it does not require time to thaw and is therefore immediately available to administer to patients. Liquid plasma retains hemostatic potential longer during storage than FFP, especially in terms of parameters associated with fibrinogen, and has been shown to decrease time to initial plasma transfusion in trauma patients, coinciding with improved adherence to balanced transfusion ratio guidelines [114], [117], [118], [119], [120]. It has been hypothesized that since liquid plasma has never gone through a freeze–thaw cycle, the protein content may be better preserved. There has only been one study evaluating the effects of liquid plasma on endothelial permeability compared to thawed FFP. This study by Cao et al. compared liquid plasma aged 0–28 days to FFP 0 and 5 days after thaw and found that liquid plasma was equivalent in mitigating TNF-α-induced permeability of an endothelial cell monolayer [121]. Although these assays did not demonstrate a difference between FFP and liquid plasma, it is possible they are not sensitive enough to detect small differences in function, and they do not fully recapitulate the complex endothelial environment *in vivo*. While liquid plasma may be particularly advantageous in prehospital or out-of-hospital settings, further studies are needed to evaluate the protective effects of liquid plasma vs. FFP on traumatic endotheliopathy and the glycocalyx.

Finally, the use of whole blood resuscitation, specifically cold-stored low-titer group O whole blood, is having a resurgence in trauma care. The Joint Trauma System has put forth guidelines on whole blood transfusion in military settings, where whole blood transfusion has been shown to improve outcomes [122], and there is growing evidence to support the use of whole blood in civilian trauma [123]. Importantly, whole blood can be administered more easily and rapidly than individual component therapy. Whole blood has demonstrated benefits in targeting the endothelial glycocalyx and vascular permeability, similar to plasma alone. In a rat model of hemorrhagic shock, whole blood and FFP demonstrated similar protective effects in restoring endothelial glycocalyx thickness to sham levels and also mitigated microvascular permeability [124], [125].

In summary, transfusion with a variety of plasma products has been shown repeatedly in preclinical studies to prevent shedding of the endothelial glycocalyx and decrease vascular permeability after hemorrhagic shock and trauma. Certain plasma proteins such as albumin and fibrinogen have been shown to mediate some of these effects, however, as mentioned previously, the full protective effects of plasma likely rely on a combination of different proteins. One area for further study is whether plasma builds the glycocalyx back in its original function and structure. Although the evidence for plasma’s effects on the endothelial glycocalyx relies heavily on *in vitro* work and animal models, two recent clinical studies suggest that plasma transfusion may indeed decrease glycocalyx shedding in patients. The first, a predefined sub-study of a randomized trial in coagulopathic critically ill patients, found that FFP transfusion reduced circulating syndecan-1 levels [126]. The second, an analysis of biomarker expression in patients in the PAMPer trial[127], found that prehospital plasma transfusion was associated with reduced syndecan-1 and thrombomodulin levels as well as multiple immune markers among the most severely injured patients [72]. Further clinical research is needed to understand how plasma transfusion affects endothelial glycocalyx shedding in patients and whether this is a potential mechanism by which plasma transfusion improves outcomes after traumatic injury. Additionally, as clinical studies are performed in this area, it is important to note the variability in plasma products that exists based on processing methods and, for single-donor plasma products, individual donor-to-donor differences. Donor-to-donor differences, for example, have been shown to produce marked variability in plasma products in terms of hemostatic potential[128] and to a lesser extent the endothelial protective effects *in vitro* and in animal models [129].

## Effects of platelet transfusion on vascular endothelial integrity

Higher platelet to RBC transfusion ratios have also been found to be associated with improved outcomes in severely injured patients with hemorrhagic shock [6], [130], [131]. A sub-study of the Pragmatic, Randomized Optimal Platelet and Plasma Ratios (PROPPR)[84] trial found that patients who received platelets were more likely to achieve hemostasis and had decreased mortality even after controlling for the volume of plasma transfusion [132]. As a result of these demonstrated benefits, similar to plasma there has recently been increased interest in whether early platelet transfusion regulates vascular endothelial integrity and endothelial glycocalyx shedding in hemorrhagic shock.

Platelets have in fact long been recognized for their role in maintaining barrier function of the microvascular endothelium through the release of growth factors or trophogens [133]. Organs perfused with platelet-rich plasma demonstrate decreased edema and protein extravasation compared to organs perfused with platelet-poor plasma [134], [135], [136], [137]. Furthermore, experimental induction of thrombocytopenia has been shown in animal models to lead to increased protein leakage, which is subsequently reversed by platelet recovery or transfusion of platelet-rich plasma [138], [139], [140]. Platelets preserve normal vascular integrity through multiple mechanisms, including the release of soluble factors (e.g. Ang-1 and S1P), preservation of tight and adherens junctions, and maintenance of endothelium ultrastructure [133], [141]. However, in multiple inflammatory conditions platelets have been found to have the opposite effect, instead promoting vascular permeability through activation of leukocytes and endothelial cells [142]. For example, in animal models of peritonitis, arthritis, or transfusion-related acute lung injury (TRALI), platelets appear to play a major role in the development of vascular permeability, and platelet depletion in these models resulted in decreased protein leakage across the endothelium [143], [144], [145]. Whether platelets are protective or mediate breakdown of the endothelial barrier may reflect the diversity of platelet contents and the degree of platelet activation in response to inflammation [145], [146], [147].

Standard apheresis platelets have indeed been shown both *in vitro* and *in vivo* in mouse models to attenuate vascular endothelial permeability after injury, however their protective effects diminish during storage. Fresh platelets decreased permeability of an endothelial monolayer in a dose-dependent fashion and attenuated VEGF-induced vascular permeability in mice; platelets stored at 22 °C for 5 days were found to be less effective [148], [149]. This platelet storage lesion is a well-known entity involving gradual loss of platelet quality in terms of metabolic function, aggregation, and morphology; the platelet storage lesion and risk of bacterial contamination with standard room temperature storage create serious limitations to platelet shelf life [150]. As a result, other platelet-based products such as freeze-dried platelets and platelet-derived extracellular vesicles (EVs) have been developed, and these products appear to be similar to platelets in reducing vascular permeability after injury. Platelet EVs and freeze-dried platelets have both been found to be equivalent to platelets in attenuating vascular permeability induced by VEGF in mice, and freeze-dried platelets additionally reduced pulmonary vascular permeability in a mouse model of hemorrhagic shock [151], [152].

**Fig. 3:**
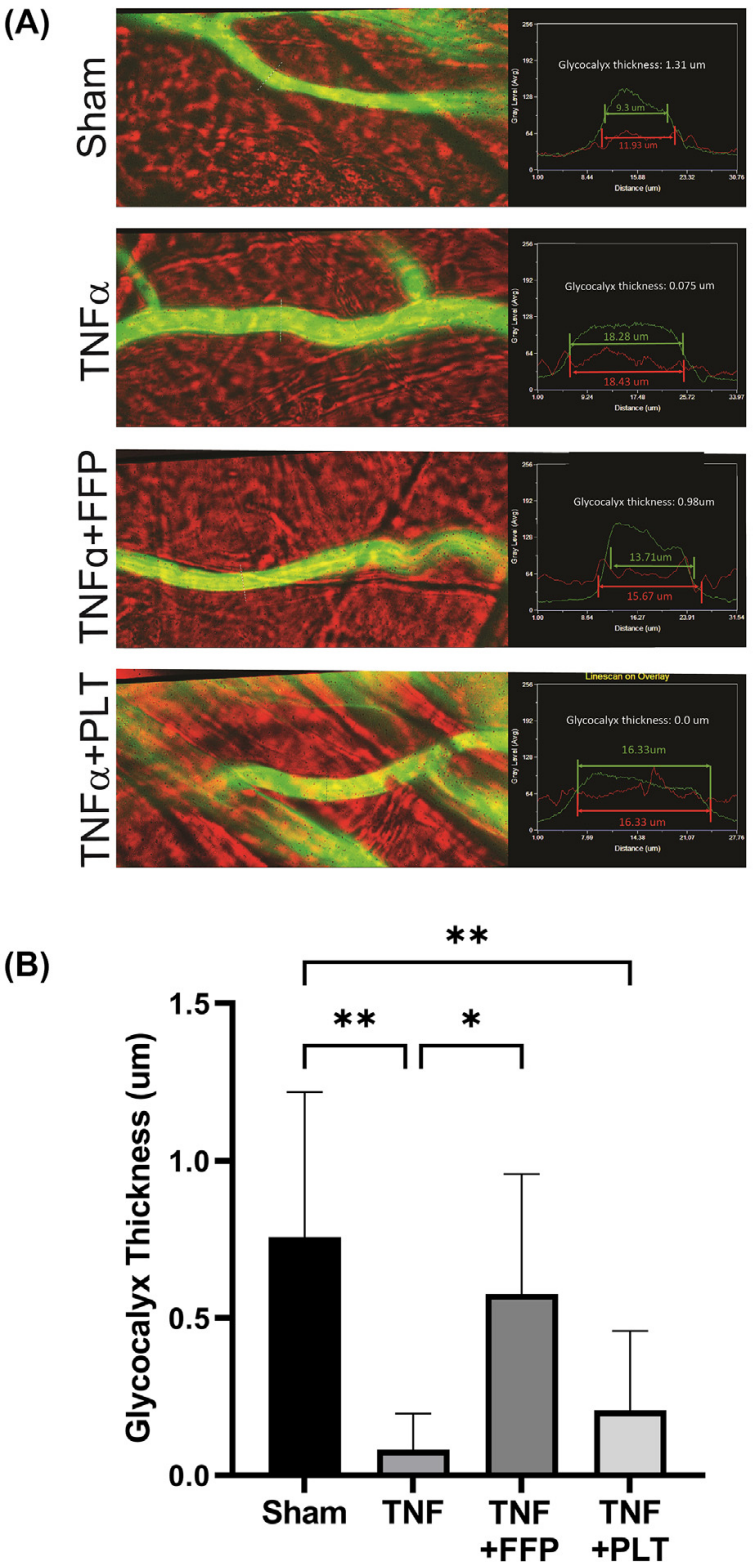
Plasma but Not Platelet Resuscitation Prevents Shedding of the Endothelial Glycocalyx. To induce vascular injury, perfusate with TNF-α (1 μg/mL) was dripped onto the cremaster prep in C57BL/6 mice and covered with thin plastic for ten minutes [153]. Temperature was maintained by dripping warm superfusate onto the plastic. At the same time as TNF-α administration, a bolus of 200 µL of fresh frozen plasma or platelets was administered via the femoral cannula. Shams were uninjured. After treatment a 100 µL bolus of 2 mg/ml FITC-tagged 70kD dextran in saline was administered via the femoral cannula. Vessels were recorded for the next 30 min. Glycocalyx thickness was determined by comparing the width of the dextran column to the width of the vessels, as 70 kD dextran is excluded from the glycocalyx layer. (A) Representative vessel images depicting glycocalyx thickness measurements. (B) Mean ± SD for each group: Sham 0.76 ± 0.46 μm, TNF-α 0.08 ± 0.11 μm, TNF-α + FFP 0.58 ± 0.38 μm, and TNF-α + PLT 0.21 ± 0.25 μm. FFP = fresh frozen plasma; PLT = platelets. *p < 0.05, **p < 0.01 by one-way ANOVA with post-hoc Tukey test.

To our knowledge, no studies have been done evaluating the effects of platelet transfusion on the endothelial glycocalyx after hemorrhagic shock. To evaluate and compare the effects of platelet and plasma transfusion on the endothelial glycocalyx after injury, our group used intravital microscopy to measure endothelial glycocalyx thickness in cremaster vessels in mice. In this experiment, after anesthesia and surgical preparation of the cremaster muscle, the cremaster was exposed to perfusate containing 1 μg/mL TNF-α to induce vascular injury. At the same time as TNF-α administration, mice were administered a bolus of 200 μL of platelets or FFP. Platelets were washed twice with saline prior to administration to remove plasma and isolate the platelet effect. A fluorescent 70 kD dextran dye was then administered intravenously to measure the thickness of the endothelial glycocalyx as previous described [153]. We found that plasma but not platelets significantly mitigated loss of the endothelial glycocalyx in this model. The mean values for cremaster vessel endothelial glycocalyx thickness in each group were as follows: Sham 0.76 μm, TNF-α 0.08 μm, TNF-α + FFP 0.58 μm, and TNF-α + platelets 0.21 μm (p < 0.001 by one-way ANOVA) (Fig. 3).

In summary, platelets contribute to the maintenance of normal vascular barrier integrity, though counterintuitively they have been found to mediate increased permeability in multiple inflammatory conditions. In pre-clinical models of hemorrhagic shock and trauma, platelet transfusion does appear to mitigate vascular leak. However, platelet products have not been shown to decrease shedding of the endothelial glycocalyx after injury, and there have been no published studies evaluating the effects of platelets transfusion on endothelial function in patients. Additionally, as noted with plasma products, individual platelet units are also subject to inter-donor variability which may impact studies. Platelets from different donors have been shown to vary in terms of both platelet aggregation and in their effects on the endothelium after hemorrhagic shock [148].

## Conclusions

The endothelial glycocalyx is a fragile but critical structure with important vasculoprotective properties. Disruption of the endothelial glycocalyx can occur rapidly in trauma and hemorrhagic shock and contributes to the EOT and ultimately multi-organ dysfunction. Plasma transfusion has the capacity to restore endothelial barrier function through a number of modalities which include preservation of tight and adherens junction proteins and decreasing inflammatory cytokines, and similarly platelets have also been found to decrease vascular permeability after injury. Furthermore, plasma transfusion prevents shedding of the endothelial glycocalyx after hemorrhagic shock and trauma in pre-clinical models, although we show in an experiment conducted for this review that platelet transfusion may not protect the glycocalyx after injury. The mechanisms by which plasma and platelets mediate their protective effects, while not fully understood, are thought to be due to soluble factors which modulate signaling pathways regulating cytoskeletal integrity and barrier function. Future directions of research will involve understanding which factors specifically produced by plasma and platelets protect the endothelium and glycocalyx in hemorrhage and trauma. Targeted therapies to rebuild the glycocalyx or stabilize the vasculature could potentially be of great benefit and synergize with plasma and platelet resuscitation in severely injured patients.

In order to mitigate endotheliopathy in the injured patient with hemorrhagic shock, this review supports the prioritization of blood product resuscitation including plasma and platelets over crystalloid fluids. This recommendation aligns with current trauma center practices of initial resuscitation with balanced ratios of standard blood products (or whole blood if available), which has been shown to improve outcomes [6], [84], [154]. Because the EOT is thought to develop within minutes after injury [11], prehospital resuscitation with plasma products or whole blood may be necessary to prevent or mitigate glycocalyx shedding and the EOT. Recent investigation into the use of prehospital blood products has been encouraging. Post hoc analyses of the PAMPer [127] and COMBAT [155] trials demonstrated that prehospital plasma had a survival benefit in patients with transport times greater than 20 min [156]. Similarly, in the military setting prehospital blood transfusions within 15 min of medical evacuation were associated with improved survival [157]. The administration of blood products with extended shelf life and without the need for thawing (e.g. never-frozen liquid plasma, whole blood, or dried products) may be more feasible than FFP-based resuscitation in the prehospital environment. In addition, in military or remote settings where resources may be limited and the logistics of frozen or even refrigerated products may be an obstacle to their delivery, the benefits of equipping medical personnel with dried plasma and platelet products–which can be stored at room temperature and rapidly reconstituted—cannot be overstated. We encourage further investigation into these products compared to standard blood products regarding their ability to target the EOT and to improve outcomes in trauma patients.

## Funding

This research did not receive any specific grant from funding agencies in the public, commercial, or not-for-profit sectors.

## CRediT authorship contribution statement

**Mark Barry:** Conceptualization, Writing – original draft, Writing – review & editing. **Shibani Pati:** Supervision, Writing – review & editing.

## Declaration of Competing Interest

The authors declare that they have no known competing financial interests or personal relationships that could have appeared to influence the work reported in this paper.

## References

[1] Haagsma J.A., Graetz N., Bolliger I., Naghavi M., Higashi H., Mullany E.C., Abera S.F., Abraham J.P., Adofo K., Alsharif U., Ameh E.A., Ammar W., Antonio C.A.T., Barrero L.H., Bekele T., Bose D., Brazinova A., Catalá-López F., Dandona L., Dandona R., Dargan P.I., De Leo D., Degenhardt L., Derrett S., Dharmaratne S.D., Driscoll T.R., Duan L., Petrovich Ermakov S., Farzadfar F., Feigin V.L., Franklin R.C., Gabbe B., Gosselin R.A., Hafezi-Nejad N., Hamadeh R.R., Hijar M., Hu G., Jayaraman S.P., Jiang G., Khader Y.S., Khan E.A., Krishnaswami S., Kulkarni C., Lecky F.E., Leung R., Lunevicius R., Lyons R.A., Majdan M., Mason-Jones A.J., Matzopoulos R., Meaney P.A., Mekonnen W., Miller T.R., Mock C.N., Norman R.E., Orozco R., Polinder S., Pourmalek F., Rahimi-Movaghar V., Refaat A., Rojas-Rueda D., Roy N., Schwebel D.C., Shaheen A., Shahraz S., Skirbekk V., Søreide K., Soshnikov S., Stein D.J., Sykes B.L., Tabb K.M., Temesgen A.M., Tenkorang E.Y., Theadom A.M., Tran B.X., Vasankari T.J., Vavilala M.S., Vlassov V.V., Woldeyohannes S.M., Yip P., Yonemoto N., Younis M.Z., Yu C., Murray C.J.L., Vos T., Balalla S., Phillips M.R. (2016). The global burden of injury: incidence, mortality, disability-adjusted life years and time trends from the Global Burden of Disease study 2013. Inj Prev..

[2] Centers for Disease Control and Prevention, National Center for Injury Prevention and Control. Web-based Injury Statistics Query and Reporting System (WISQARS) 2017 [Available from: www.cdc.gov/injury/wisqars.

[3] Kauvar D.S., Wade C.E. (2005). The epidemiology and modern management of traumatic hemorrhage: US and international perspectives. Crit. Care.

[4] Mitra B., Gabbe B.J., Kaukonen K.M., Olaussen A., Cooper D.J., Cameron P.A. (2014). Long-term outcomes of patients receiving a massive transfusion after trauma. Shock..

[5] Holcomb J.B., Jenkins D., Rhee P., Johannigman J., Mahoney P., Mehta S., Cox E.D., Gehrke M.J., Beilman G.J., Schreiber M., Flaherty S.F., Grathwohl K.W., Spinella P.C., Perkins J.G., Beekley A.C., McMullin N.R., Park M.S., Gonzalez E.A., Wade C.E., Dubick M.A., Schwab C.W., Moore F.A., Champion H.R., Hoyt D.B., Hess J.R. (2007). Damage control resuscitation: directly addressing the early coagulopathy of trauma. J. Trauma..

[6] Cannon J.W., Khan M.A., Raja A.S., Cohen M.J., Como J.J., Cotton B.A., Dubose J.J., Fox E.E., Inaba K., Rodriguez C.J., Holcomb J.B., Duchesne J.C. (2017). Damage control resuscitation in patients with severe traumatic hemorrhage: A practice management guideline from the Eastern Association for the Surgery of Trauma. J. Trauma Acute Care Surg..

[7] Longo D.L., Cannon J.W. (2018). Hemorrhagic shock. N. Engl. J. Med..

[8] Holcomb J.B., Pati S. (2013). Optimal trauma resuscitation with plasma as the primary resuscitative fluid: the surgeon's perspective. Hematol. Am. Soc. Hematol. Educ. Program..

[9] Jenkins D.H., Rappold J.F., Badloe J.F., Berséus O., Blackbourne C.L., Brohi K.H., Butler F.K., Cap L.A.P., Cohen M.J., Davenport R., DePasquale M., Doughty H., Glassberg E., Hervig T., Hooper T.J., Kozar R., Maegele M., Moore E.E., Murdock A., Ness P.M., Pati S., Rasmussen C.T., Sailliol A., Schreiber M.A., Sunde G.A., van de Watering L.M.G., Ward K.R., Weiskopf R.B., White N.J., Strandenes G., Spinella P.C. (2014). Trauma hemostasis and oxygenation research position paper on remote damage control resuscitation: definitions, current practice, and knowledge gaps. Shock..

[10] White N.J., Ward K.R., Pati S., Strandenes G., Cap A.P. (2017). Hemorrhagic blood failure: Oxygen debt, coagulopathy, and endothelial damage. J. Trauma Acute Care Surg..

[11] Naumann D.N., Hazeldine J., Davies D.J., Bishop J., Midwinter M.J., Belli A., Harrison P., Lord J.M. (2018). Endotheliopathy of trauma is an on-scene phenomenon, and is associated with multiple organ dysfunction syndrome: a prospective observational study. Shock..

[12] Alphonsus C.S., Rodseth R.N. (2014). The endothelial glycocalyx: a review of the vascular barrier. Anaesthesia..

[13] Li L., Bonventre J.V. (2016). Endothelial glycocalyx: not just a sugar coat. Am. J. Respir. Crit. Care Med..

[14] Pries A.R., Secomb T.W., Gaehtgens P. (2000). The endothelial surface layer. Pflugers Arch..

[15] Reitsma S., Slaaf D.W., Vink H., van M.A., Zandvoort, Oude Egbrink M.G. (2007). The endothelial glycocalyx: composition, functions, and visualization. Pflugers Arch..

[16] Jedlicka J., Becker B.F., Chappell D. (2020). Endothelial glycocalyx. Crit. Care Clin..

[17] Zhang X., Sun D., Song J.W., Zullo J., Lipphardt M., Coneh-Gould L., Goligorsky M.S. (2018). Endothelial cell dysfunction and glycocalyx - A vicious circle. Matrix Biol..

[18] Tarbell J.M., Pahakis M.Y. (2006). Mechanotransduction and the glycocalyx. J. Intern. Med..

[19] Lipowsky H.H. (2005). Microvascular rheology and hemodynamics. Microcirculation..

[20] Butler M.J., Down C.J., Foster R.R., Satchell S.C. (2020). The pathological relevance of increased endothelial glycocalyx permeability. Am. J. Pathol..

[21] Rahbar E., Cardenas J.C., Baimukanova G., Usadi B., Bruhn R., Pati S., Ostrowski S.R., Johansson P.I., Holcomb J.B., Wade C.E. (2015). Endothelial glycocalyx shedding and vascular permeability in severely injured trauma patients. J. Transl. Med..

[22] Becker B.F., Chappell D., Bruegger D., Annecke T., Jacob M. (2010). Therapeutic strategies targeting the endothelial glycocalyx: acute deficits, but great potential. Cardiovasc. Res..

[23] Becker B.F., Jacob M., Leipert S., Salmon A.H., Chappell D. (2015). Degradation of the endothelial glycocalyx in clinical settings: searching for the sheddases. Br. J. Clin. Pharmacol..

[24] Cabrales P., Vázquez B.Y., Tsai A.G., Intaglietta M. (2007). Microvascular and capillary perfusion following glycocalyx degradation. J. Appl. Physiol. (1985).

[25] Belousoviene E., Kiudulaite I., Pilvinis V., Pranskunas A. (2021). Links between endothelial glycocalyx changes and microcirculatory parameters in septic patients. Life (Basel).

[26] Rienks M., Carai P., van Teeffelen J., Eskens B., Verhesen W., Hemmeryckx B., Johnson D.M., van Leeuwen R., Jones E.A., Heymans S., Papageorgiou A.-P. (2018). SPARC preserves endothelial glycocalyx integrity, and protects against adverse cardiac inflammation and injury during viral myocarditis. Matrix Biol..

[27] Uchimido R., Schmidt E.P., Shapiro N.I. (2019). The glycocalyx: a novel diagnostic and therapeutic target in sepsis. Crit. Care.

[28] Goligorsky M.S., Sun D. (2020). Glycocalyx in endotoxemia and sepsis. Am. J. Pathol..

[29] Manon-Jensen T., Multhaupt H.A., Couchman J.R. (2013). Mapping of matrix metalloproteinase cleavage sites on syndecan-1 and syndecan-4 ectodomains. FEBS J..

[30] Chappell D., Jacob M., Rehm M., Stoeckelhuber M., Welsch U., Conzen P. (2008). Heparinase selectively sheds heparan sulphate from the endothelial glycocalyx. Biol. Chem..

[31] Manon-Jensen T., Itoh Y., Couchman J.R. (2010). Proteoglycans in health and disease: the multiple roles of syndecan shedding. FEBS J..

[32] Schmidt E.P., Yang Y., Janssen W.J., Gandjeva A., Perez M.J., Barthel L., Zemans R.L., Bowman J.C., Koyanagi D.E., Yunt Z.X., Smith L.P., Cheng S.S., Overdier K.H., Thompson K.R., Geraci M.W., Douglas I.S., Pearse D.B., Tuder R.M. (2012). The pulmonary endothelial glycocalyx regulates neutrophil adhesion and lung injury during experimental sepsis. Nat. Med..

[33] Ramnath R., Foster R.R., Qiu Y., Cope G., Butler M.J., Salmon A.H., Mathieson P.W., Coward R.J., Welsh G.I., Satchell S.C. (2014). Matrix metalloproteinase 9-mediated shedding of syndecan 4 in response to tumor necrosis factor α: a contributor to endothelial cell glycocalyx dysfunction. FASEB J..

[34] Astapenko D., Benes J., Pouska J., Lehmann C., Islam S., Cerny V. (2019). Endothelial glycocalyx in acute care surgery - what anaesthesiologists need to know for clinical practice. BMC Anesthesiol..

[35] Diebel L.N., Diebel M.E., Martin J.V., Liberati D.M. (2018). Acute hyperglycemia exacerbates trauma-induced endothelial and glycocalyx injury: An in vitro model. J. Trauma Acute Care Surg..

[36] Johansson P.I., Stensballe J., Ostrowski S.R. (2017). Shock induced endotheliopathy (SHINE) in acute critical illness - a unifying pathophysiologic mechanism. Crit. Care.

[37] Carge M.J., Liberati D.M., Diebel L.N. (2021). A biomimetic shock model on the effect of endothelial aging on vascular barrier properties. J Trauma Acute Care Surg..

[38] Martin J.V., Liberati D.M., Diebel L.N. (2017). Disparate effects of catecholamines under stress conditions on endothelial glycocalyx injury: An in vitro model. Am. J. Surg..

[39] Chignalia A.Z., Yetimakman F., Christiaans S.C., Unal S., Bayrakci B., Wagener B.M., Russell R.T., Kerby J.D., Pittet J.-F., Dull R.O. (2016). The glycocalyx and trauma: a review. Shock..

[40] Ostrowski S.R., Johansson P.I. (2012). Endothelial glycocalyx degradation induces endogenous heparinization in patients with severe injury and early traumatic coagulopathy. J. Trauma Acute Care Surg..

[41] Haywood-Watson R.J., Holcomb J.B., Gonzalez E.A., Peng Z., Pati S., Park P.W. (2011). Modulation of syndecan-1 shedding after hemorrhagic shock and resuscitation. PLoS One..

[42] Halbgebauer R., Braun C.K., Denk S., Mayer B., Cinelli P., Radermacher P., Wanner G.A., Simmen H.-P., Gebhard F., Rittirsch D., Huber-Lang M. (2018). Hemorrhagic shock drives glycocalyx, barrier and organ dysfunction early after polytrauma. J. Crit. Care.

[43] Potter D.R., Baimukanova G., Keating S.M., Deng X., Chu J.A., Gibb S.L., Peng Z., Muench M.O., Fomin M.E., Spinella P.C., Kozar R., Pati S. (2015). Fresh frozen plasma and spray-dried plasma mitigate pulmonary vascular permeability and inflammation in hemorrhagic shock. J. Trauma Acute Care Surg..

[44] Claesson-Welsh L., Dejana E., McDonald D.M. (2021). Permeability of the endothelial barrier: identifying and reconciling controversies. Trends Mol. Med..

[45] Galley H.F., Webster N.R. (2004). Physiology of the endothelium. Br. J. Anaesth..

[46] Sieve I., Munster-Kuhnel A.K., Hilfiker-Kleiner D. (2018). Regulation and function of endothelial glycocalyx layer in vascular diseases. Vasc.Pharmacol..

[47] Johansson P.I., Sørensen A., Perner A., Welling K., Wanscher M., Larsen C.F., Ostrowski S.R. (2011). Disseminated intravascular coagulation or acute coagulopathy of trauma shock early after trauma? An observational study. Crit. Care.

[48] Liao J.K. (2013). Linking endothelial dysfunction with endothelial cell activation. J. Clin. Invest..

[49] Michiels C., Arnould T., Remacle J. (2000). Endothelial cell responses to hypoxia: initiation of a cascade of cellular interactions. Biochim. Biophys. Acta, Gen. Subj..

[50] Kang S., Kishimoto T. (2021). Interplay between interleukin-6 signaling and the vascular endothelium in cytokine storms. Exp. Mol. Med..

[51] Pober J.S. (2002). Endothelial activation: intracellular signaling pathways. Arthritis Res..

[52] Minami T., Sugiyama A., Wu S.Q., Abid R., Kodama T., Aird W.C. (2004). Thrombin and phenotypic modulation of the endothelium. Arterioscler. Thromb. Vasc. Biol..

[53] Knop M., Gerke V. (2002). Ca2+ -regulated secretion of tissue-type plasminogen activator and von Willebrand factor in human endothelial cells. Biochim. Biophys. Acta, Gen. Subj..

[54] Moore E.E., Moore H.B., Kornblith L.Z., Neal M.D., Hoffman M., Mutch N.J., Schöchl H., Hunt B.J., Sauaia A. (2021). Trauma-induced coagulopathy. Nat. Rev. Dis. Primers.

[55] Abdullah S., Karim M., Legendre M., Rodriguez L., Friedman J., Cotton-Betteridge A., Drury R., Packer J., Guidry C., Duchesne J., Taghavi S., Jackson-Weaver O. (2021). Hemorrhagic shock and resuscitation causes glycocalyx shedding and endothelial oxidative stress preferentially in the lung and intestinal vasculature. Shock..

[56] Mathis S., Putzer G., Schneeberger S., Martini J. (2021). The endothelial glycocalyx and organ preservation-from physiology to possible clinical implications for solid organ transplantation. Int. J. Mol. Sci..

[57] Bongoni A.K., Lu B., McRae J.L., Salvaris E.J., Toonen E.J.M., Vikstrom I. (2019). Complement-mediated damage to the glycocalyx plays a role in renal ischemia-reperfusion injury in mice. Transplant Direct..

[58] Xu C., Chang A., Hack B.K., Eadon M.T., Alper S.L., Cunningham P.N. (2014). TNF-mediated damage to glomerular endothelium is an important determinant of acute kidney injury in sepsis. Kidney Int..

[59] Yau J.W., Teoh H., Verma S. (2015). Endothelial cell control of thrombosis. BMC Cardiovasc. Disord..

[60] Kazmi R.S., Boyce S., Lwaleed B.A. (2015). Homeostasis of hemostasis: the role of endothelium. Semin. Thromb. Hemost..

[61] Pillai V.G., Bao J., Zander C.B., McDaniel J.K., Chetty P.S., Seeholzer S.H., Bdeir K., Cines D.B., Zheng X.L. (2016). Human neutrophil peptides inhibit cleavage of von Willebrand factor by ADAMTS13: a potential link of inflammation to TTP. Blood.

[62] Bdeir K., Murciano J.-C., Tomaszewski J., Koniaris L., Martinez J., Cines D.B., Muzykantov V.R., Higazi A.-R. (2000). Urokinase mediates fibrinolysis in the pulmonary microvasculature. Blood.

[63] Chapman M.P., Moore E.E., Moore H.B., Gonzalez E., Gamboni F., Chandler J.G., Mitra S., Ghasabyan A., Chin T.L., Sauaia A., Banerjee A., Silliman C.C. (2016). Overwhelming tPA release, not PAI-1 degradation, is responsible for hyperfibrinolysis in severely injured trauma patients. J. Trauma Acute Care Surg..

[64] Brouns S.L.N., Provenzale I., van Geffen J.P., van der Meijden P.E.J., Heemskerk J.W.M. (2020). Localized endothelial-based control of platelet aggregation and coagulation under flow: A proof-of-principle vessel-on-a-chip study. J. Thromb. Haemost..

[65] Vink H., Constantinescu A.A., Spaan J.A. (2000). Oxidized lipoproteins degrade the endothelial surface layer : implications for platelet-endothelial cell adhesion. Circulation.

[66] Chang R., Cardenas J.C., Wade C.E., Holcomb J.B. (2016). Advances in the understanding of trauma-induced coagulopathy. Blood.

[67] Britten M.W., Lümers L., Tominaga K., Peters J., Dirkmann D. (2021). Glycocalyx components affect platelet function, whole blood coagulation, and fibrinolysis: an in vitro study suggesting a link to trauma-induced coagulopathy. BMC Anesthesiol..

[68] Gonzalez Rodriguez E., Ostrowski S.R., Cardenas J.C., Baer L.A., Tomasek J.S., Henriksen H.H., Stensballe J., Cotton B.A., Holcomb J.B., Johansson P.I., Wade C.E. (2017). Syndecan-1: a quantitative marker for the endotheliopathy of trauma. J. Am. Coll. Surg..

[69] Johansson P.I., Stensballe J., Rasmussen L.S., Ostrowski S.R. (2011). A high admission syndecan-1 level, a marker of endothelial glycocalyx degradation, is associated with inflammation, protein C depletion, fibrinolysis, and increased mortality in trauma patients. Ann. Surg..

[70] Johansson P.I., Henriksen H.H., Stensballe J., Gybel-Brask M., Cardenas J.C., Baer L.A., Cotton B.A., Holcomb J.B., Wade C.E., Ostrowski S.R. (2017). Traumatic endotheliopathy: a prospective observational study of 424 severely injured patients. Ann. Surg..

[71] Gonzalez Rodriguez E., Cardenas J.C., Cox C.S., Kitagawa R.S., Stensballe J., Holcomb J.B., Johansson P.I., Wade C.E. (2018). Traumatic brain injury is associated with increased syndecan-1 shedding in severely injured patients. Scand. J. Trauma Resusc. Emerg. Med..

[72] Gruen D.S., Brown J.B., Guyette F.X., Vodovotz Y., Johansson P.I., Stensballe J., Barclay D.A., Yin J., Daley B.J., Miller R.S., Harbrecht B.G., Claridge J.A., Phelan H.A., Neal M.D., Zuckerbraun B.S., Billiar T.R., Sperry J.L. (2020). Prehospital plasma is associated with distinct biomarker expression following injury. JCI Insight..

[73] Wei S., Gonzalez Rodriguez E., Chang R., Holcomb J.B., Kao L.S., Wade C.E., Holcomb J.B., Wade C.E., del Junco D.J., Fox E.E., Matijevic N., Podbielski J., Beeler A.M., Tilley B.C., Baraniuk S., Zhu H., Nixon J., Seay R., Appana S.N., Yang H., Gonzalez M.O., Baer L., Willa Wang Y.-W., Hula B.S., Espino E., Nguyen A.n., Pawelczyk N., Arora-Nutall K.D., Sharma R., Cardenas J.C., Rahbar E., Burnett T., Clark D., van Belle G., May S., Leroux B., Hoyt D., Powell J., Sheehan K., Hubbard A., Arkin A.P., Hess J.R., Callum J., Cotton B.A., Vincent L., Welch T., Poole T., Pivalizza E.G., Gumbert S.D., Bai Y.u., McCarthy J.J., Noland A., Hobbs R., Bulger E.M., Klotz P., Cattin L., Warner K.J., Wilson A., Boman D., White N., Grabinsky A., Daniel-Johnson J.A., Cohen M.J., Callcut R.A., Nelson M., Redick B., Conroy A., Steurer M.P., Maxim P.C., Fiebig E., Moore J., Mallari E., Muskat P., Johannigman J.A., Robinson B.RH., Branson R.D., Gomaa D., Barczak C., Bennett S., Carey P.M., Miller C.N., Hancock H., Rodriguez C., Inaba K., Zhu J.G., Wong M.D., Menchine M., Katzberg K., Henderson S.O., McKeever R., Shulman I.A., Nelson J.M., Tuma C.W., Matsushita C.Y., Scalea T.M., Stein D.M., Shaffer C.K., Wade C., Herrera A.V., Kallam S., Wade S.E., Galvagno S.M., Fontaine M.J., Hunt J.M., Cooke R.K., Fabian T.C., Weinberg J.A., Croce M.A., Wilson S., Panzer-Baggett S., Waddle-Smith L., Flax S., Brasel K.J., Walsh P., Milia D., Nelson A., Kaslow O., Aufderheide T.P., Gottschall J.L., Carpenter E., O'Keeffe T., Rokowski L.L., Denninghoff K.R., Redford D.T., Novak D.J., Knoll S., Kerby J.D., Pittet J.-F., Bosarge P.L., Pierce A.T., Williams C.R., Stephens S.W., Wang H.E., Marques M.B., Schreiber M.A., Watters J.M., Underwood S.J., Groat T., Newgard C., Merkel M., Scanlan R.M., Miller B., Rizoli S., Tien H., Nascimento B., Trpcic S., Sobrian-Couroux S., Reis M., Pérez A., Belo S.E., Merkley L., Colavecchia C. (2018). Elevated Syndecan-1 after Trauma and Risk of Sepsis: A Secondary Analysis of Patients from the Pragmatic, Randomized Optimal Platelet and Plasma Ratios (PROPPR) Trial. J. Am. Coll. Surg..

[74] Ostrowski S.R., Haase N., Müller R.B., Møller M.H., Pott F.C., Perner A., Johansson P.I. (2015). Association between biomarkers of endothelial injury and hypocoagulability in patients with severe sepsis: a prospective study. Crit. Care.

[75] Neves F.M., Meneses G.C., Sousa N.E., Menezes R.R., Parahyba M.C., Martins A.M. (2015). Syndecan-1 in acute decompensated heart failure-association with renal function and mortality. Circ. J..

[76] Padberg J.-S., Wiesinger A., di Marco G.S., Reuter S., Grabner A., Kentrup D., Lukasz A., Oberleithner H., Pavenstädt H., Brand M., Kümpers P. (2014). Damage of the endothelial glycocalyx in chronic kidney disease. Atherosclerosis..

[77] Dogné S., Flamion B., Caron N. (2018). Endothelial glycocalyx as a shield against diabetic vascular complications: involvement of hyaluronan and hyaluronidases. Arterioscler. Thromb. Vasc. Biol..

[78] Welling H., Henriksen H.H., Gonzalez-Rodriguez E.R., Stensballe J., Huzar T.F., Johansson P.I., Wade C.E. (2020). Endothelial glycocalyx shedding in patients with burns. Burns..

[79] Teng Y.H., Aquino R.S., Park P.W. (2012). Molecular functions of syndecan-1 in disease. Matrix Biol..

[80] BioGPS Dataset: GeneAtlas U133A, gcrma. Syndecan-1 (SDC1). http://biogps.org/#goto=genereport&id=6382. Accessed February 3, 2022.

[81] Naumann D.N., Hazeldine J., Midwinter M.J., Hutchings S.D., Harrison P. (2018). Poor microcirculatory flow dynamics are associated with endothelial cell damage and glycocalyx shedding after traumatic hemorrhagic shock. J. Trauma Acute Care Surg..

[82] Wu Q., Gao W., Zhou J., He G., Ye J., Fang F., Luo J., Wang M., Xu H., Wang W. (2019). Correlation between acute degradation of the endothelial glycocalyx and microcirculation dysfunction during cardiopulmonary bypass in cardiac surgery. Microvasc. Res..

[83] Chang R., Holcomb J.B. (2017). Optimal fluid therapy for traumatic hemorrhagic shock. Crit. Care Clin..

[84] Holcomb J.B., Tilley B.C., Baraniuk S., Fox E.E., Wade C.E., Podbielski J.M., del Junco D.J., Brasel K.J., Bulger E.M., Callcut R.A., Cohen M.J., Cotton B.A., Fabian T.C., Inaba K., Kerby J.D., Muskat P., O’Keeffe T., Rizoli S., Robinson B.R.H., Scalea T.M., Schreiber M.A., Stein D.M., Weinberg J.A., Callum J.L., Hess J.R., Matijevic N., Miller C.N., Pittet J.-F., Hoyt D.B., Pearson G.D., Leroux B., van Belle G. (2015). Transfusion of plasma, platelets, and red blood cells in a 1:1:1 vs a 1:1:2 ratio and mortality in patients with severe trauma: the PROPPR randomized clinical trial. JAMA.

[85] Barelli S., Alberio L. (2018). The role of plasma transfusion in massive bleeding: protecting the endothelial glycocalyx?. Front. Med. (Lausanne)..

[86] Pati S., Matijevic N., Doursout M.-F., Ko T., Cao Y., Deng X., Kozar R.A., Hartwell E., Conyers J., Holcomb J.B. (2010). Protective effects of fresh frozen plasma on vascular endothelial permeability, coagulation, and resuscitation after hemorrhagic shock are time dependent and diminish between days 0 and 5 after thaw. J. Trauma.

[87] Letourneau P.A., Pati S., Gerber M.H., Jimenez F., Holcomb J.B. (2010). Fresh frozen plasma increases adhesion molecule expression on human pulmonary endothelial cells. J. Surg. Res..

[88] Kozar R.A., Peng Z., Zhang R., Holcomb J.B., Pati S., Park P., Ko T.C., Paredes A. (2011). Plasma restoration of endothelial glycocalyx in a rodent model of hemorrhagic shock. Anesth. Analg..

[89] Peng Z., Pati S., Potter D., Brown R., Holcomb J.B., Grill R., Wataha K., Park P.W., Xue H., Kozar R.A. (2013). Fresh frozen plasma lessens pulmonary endothelial inflammation and hyperpermeability after hemorrhagic shock and is associated with loss of syndecan 1. Shock..

[90] Torres L.N., Sondeen J.L., Ji L., Dubick M.A., Torres F.I. (2013). Evaluation of resuscitation fluids on endothelial glycocalyx, venular blood flow, and coagulation function after hemorrhagic shock in rats. J. Trauma Acute Care Surg..

[91] Duong C.N., Vestweber D. (2020). Mechanisms ensuring endothelial junction integrity beyond VE-cadherin. Front. Physiol..

[92] Giannotta M., Trani M., Dejana E. (2013). VE-cadherin and endothelial adherens junctions: active guardians of vascular integrity. Dev. Cell.

[93] Dejana E., Orsenigo F., Lampugnani M.G. (2008). The role of adherens junctions and VE-cadherin in the control of vascular permeability. J. Cell Sci..

[94] Barry M., Trivedi A., Miyazawa B.Y., Vivona L.R., Khakoo M., Zhang H., Pathipati P., Bagri A., Gatmaitan M.G., Kozar R., Stein D., Pati S. (2021). Cryoprecipitate attenuates the endotheliopathy of trauma in mice subjected to hemorrhagic shock and trauma. J Trauma Acute Care Surg..

[95] Pati S., Peng Z., Wataha K., Miyazawa B., Potter D.R., Kozar R.A. (2018). Lyophilized plasma attenuates vascular permeability, inflammation and lung injury in hemorrhagic shock. PLoS One.

[96] Schenk S., Schoenhals G.J., de Souza G., Mann M. (2008). A high confidence, manually validated human blood plasma protein reference set. BMC Med. Genomics.

[97] Deng X., Cao Y., Huby M.P., Duan C., Baer L., Peng Z., Kozar R.A., Doursout M.-F., Holcomb J.B., Wade C.E., Ko T.C. (2016). Adiponectin in fresh frozen plasma contributes to restoration of vascular barrier function after hemorrhagic shock. Shock..

[98] Alves N.G., Trujillo A.N., Breslin J.W., Yuan S.Y. (2019). Sphingosine-1-phosphate reduces hemorrhagic shock and resuscitation-induced microvascular leakage by protecting endothelial mitochondrial integrity. Shock..

[99] Aldecoa C., Llau J.V., Nuvials X., Artigas A. (2020). Role of albumin in the preservation of endothelial glycocalyx integrity and the microcirculation: a review. Ann. Intensive Care..

[100] Diebel M.E., Diebel L.N., Liberati D.M. (2019). Protective effects of plasma products on the endothelial-glycocalyx barrier following trauma-hemorrhagic shock: Is sphingosine-1 phosphate responsible?. J. Trauma Acute Care Surg..

[101] Trieu M., van Meurs M., van Leeuwen A.L.I., Van Slyke P., Hoang V., Geeraedts L.M.G., Boer C., van den Brom C.E. (2018). Vasculotide, an angiopoietin-1 mimetic, restores microcirculatory perfusion and microvascular leakage and decreases fluid resuscitation requirements in hemorrhagic shock. Anesthesiology.

[102] Childs E.W., Tharakan B., Byrge N., Tinsley J.H., Hunter F.A., Smythe W.R. (2008). Angiopoietin-1 inhibits intrinsic apoptotic signaling and vascular hyperpermeability following hemorrhagic shock. Am. J. Physiol. Heart Circ. Physiol..

[103] Pati S., Potter D.R., Baimukanova G., Farrel D.H., Holcomb J.B., Schreiber M.A. (2016). Modulating the endotheliopathy of trauma: factor concentrate versus fresh frozen plasma. J. Trauma Acute Care Surg..

[104] Ferrer R., Mateu X., Maseda E., Yébenes J.C., Aldecoa C., De Haro C., Ruiz-Rodriguez J.C., Garnacho-Montero J. (2018). Non-oncotic properties of albumin. A multidisciplinary vision about the implications for critically ill patients. Expert Rev. Clin. Pharmacol..

[105] Torres L.N., Chung K.K., Salgado C.L., Dubick M.A., Torres Filho I.P. (2017). Low-volume resuscitation with normal saline is associated with microvascular endothelial dysfunction after hemorrhage in rats, compared to colloids and balanced crystalloids. Crit. Care.

[106] Horstick G., Lauterbach M., Kempf T., Bhakdi S., Heimann A., Horstick M., Meyer J., Kempski O. (2002). Early albumin infusion improves global and local hemodynamics and reduces inflammatory response in hemorrhagic shock. Crit. Care Med..

[107] Hayakawa M., Gando S., Ono Y., Wada T., Yanagida Y., Sawamura A. (2015). Fibrinogen level deteriorates before other routine coagulation parameters and massive transfusion in the early phase of severe trauma: a retrospective observational study. Semin. Thromb. Hemost..

[108] Wu F., Kozar R.A. (2019). Fibrinogen protects against barrier dysfunction through maintaining cell surface syndecan-1 in vitro. Shock..

[109] Chipman A.M., Wu F., Kozar R.A. (2021). Fibrinogen inhibits microRNA-19b, a novel mechanism for repair of haemorrhagic shock-induced endothelial cell dysfunction. Blood Transfus..

[110] Wu F., Chipman A., Dong J.-F., Kozar R.A. (2021). Fibrinogen activates PAK1/cofilin signaling pathway to protect endothelial barrier integrity. Shock.

[111] Wu F., Chipman A., Pati S., Miyasawa B., Corash L., Kozar R.A. (2020). Resuscitative strategies to modulate the endotheliopathy of trauma: from cell to patient. Shock..

[112] Pusateri A.E., Given M.B., Schreiber M.A., Spinella P.C., Pati S., Kozar R.A. (2016). Dried plasma: state of the science and recent developments. Transfusion..

[113] Pusateri A.E., Butler F.K., Shackelford S.A., Sperry J.L., Moore E.E., Cap A.P., Taylor A.L., Homer M.J., Hoots W.K., Weiskopf R.B., Davis M.R. (2019). The need for dried plasma - a national issue. Transfusion..

[114] Matijevic N., Kostousov V., Wang Y.-W., Wade C.E., Wang W., Letourneau P., Hartwell E., Kozar R., Ko T., Holcomb J.B. (2011). Multiple levels of degradation diminish hemostatic potential of thawed plasma. J. Trauma.

[115] Garrigue D., Godier A., Glacet A., Labreuche J., Kipnis E., Paris C., Duhamel A., Resch E., Bauters A., Machuron F., Renom P., Goldstein P., Tavernier B., Sailliol A., Susen S. (2018). French lyophilized plasma versus fresh frozen plasma for the initial management of trauma-induced coagulopathy: a randomized open-label trial. J. Thromb. Haemost..

[116] Wataha K., Menge T., Deng X., Shah A., Bode A., Holcomb J.B., Potter D., Kozar R., Spinella P.C., Pati S. (2013). Spray-dried plasma and fresh frozen plasma modulate permeability and inflammation in vitro in vascular endothelial cells. Transfusion..

[117] Beattie G., Cohan C.M., Ng V.L., Victorino G.P. (2020). Liquid plasma: A solution to optimizing early and balanced plasma resuscitation in massive transfusion. J. Trauma Acute Care Surg..

[118] Chehab M., Ditillo M., Obaid O., Nelson A., Poppe B., Douglas M., Anand T., Bible L., Joseph B. (2021). Never-frozen liquid plasma transfusion in civilian trauma: a nationwide propensity-matched analysis. J. Trauma Acute Care Surg..

[119] Matijevic N., Wang Y.-W., Cotton B.A., Hartwell E., Barbeau J.M., Wade C.E., Holcomb J.B. (2013). Better hemostatic profiles of never-frozen liquid plasma compared with thawed fresh frozen plasma. J Trauma Acute Care Surg..

[120] Meledeo M.A., Peltier G.C., McIntosh C.S., Bynum J.A., Corley J.B., Cap A.P. (2021). Coagulation function of never frozen liquid plasma stored for 40 days. Transfusion..

[121] Cao Y., Dua A., Matijevic N., Wang Y.-W., Pati S., Wade C.E., Ko T.C., Holcomb J.B. (2014). Never-frozen liquid plasma blocks endothelial permeability as effectively as thawed fresh frozen plasma. J. Trauma Acute Care Surg..

[122] Shackelford S.A., Gurney J.M., Taylor A.L., Keenan S., Corley J.B., Cunningham C.W., Drew B.G., Jensen S.D., Kotwal R.S., Montgomery H.R., Nance E.T., Remley M.A., Cap A.P. (2021). Joint trauma system, defense committee on trauma, and armed services blood program consensus statement on whole blood. Transfusion..

[123] Leeper C.M., Yazer M.H., Neal M.D. (2020). Whole-blood resuscitation of injured patients: innovating from the past. JAMA Surg..

[124] Torres Filho I.P., Torres L.N., Salgado C., Dubick M.A. (2016). Plasma syndecan-1 and heparan sulfate correlate with microvascular glycocalyx degradation in hemorrhaged rats after different resuscitation fluids. Am. J. Physiol. Heart Circ. Physiol..

[125] Torres L.N., Sondeen J.L., Dubick M.A., Torres F.I. (2014). Systemic and microvascular effects of resuscitation with blood products after severe hemorrhage in rats. J. Trauma Acute Care Surg..

[126] Straat M., Müller M.CA., Meijers J.CM., Arbous M.S., Spoelstra - de Man A.ME., Beurskens C.JP., Vroom M.B., Juffermans N.P. (2015). Effect of transfusion of fresh frozen plasma on parameters of endothelial condition and inflammatory status in non-bleeding critically ill patients: a prospective substudy of a randomized trial. Crit. Care.

[127] Sperry J.L., Guyette F.X., Brown J.B., Yazer M.H., Triulzi D.J., Early-Young B.J., Adams P.W., Daley B.J., Miller R.S., Harbrecht B.G., Claridge J.A., Phelan H.A., Witham W.R., Putnam A.T., Duane T.M., Alarcon L.H., Callaway C.W., Zuckerbraun B.S., Neal M.D., Rosengart M.R., Forsythe R.M., Billiar T.R., Yealy D.M., Peitzman A.B., Zenati M.S. (2018). Prehospital plasma during air medical transport in trauma patients at risk for hemorrhagic shock. N. Engl. J. Med..

[128] Spinella P.C., Frazier E., Pidcoke H.F., Dietzen D.J., Pati S., Gorkun O., Aden J.K., Norris P.J., Cap A.P. (2015). All plasma products are not created equal: characterizing differences between plasma products. J. Trauma Acute Care Surg..

[129] Chipman A.M., Pati S., Potter D., Wu F., Lin M., Kozar R.A. (2020). Is all plasma created equal? A pilot study of the effect of interdonor variability. J. Trauma Acute Care Surg..

[130] Holcomb J.B., Wade C.E., Michalek J.E., Chisholm G.B., Zarzabal L.A., Schreiber M.A., Gonzalez E.A., Pomper G.J., Perkins J.G., Spinella P.C., Williams K.L., Park M.S. (2008). Increased plasma and platelet to red blood cell ratios improves outcome in 466 massively transfused civilian trauma patients. Ann. Surg..

[131] Holcomb J.B., Zarzabal L.A., Michalek J.E., Kozar R.A., Spinella P.C., Perkins J.G., Matijevic N., Dong J.-F., Pati S., Wade C.E. (2011). Increased platelet:RBC ratios are associated with improved survival after massive transfusion. J. Trauma.

[132] Cardenas J.C., Zhang X.u., Fox E.E., Cotton B.A., Hess J.R., Schreiber M.A., Wade C.E., Holcomb J.B. (2018). Platelet transfusions improve hemostasis and survival in a substudy of the prospective, randomized PROPPR trial. Blood Adv..

[133] Ho-Tin-Noe B., Demers M., Wagner D.D. (2011). How platelets safeguard vascular integrity. J. Thrombosis Haemostasis: JTH.

[134] Danielli J.F. (1940). Capillary permeability and oedema in the perfused frog. J. Physiol..

[135] Gimbrone M.A., Aster R.H., Cotran R.S., Corkery J., Jandl J.H., Folkman J. (1969). Preservation of vascular integrity in organs perfused in vitro with a platelet-rich medium. Nature.

[136] McDonagh P.F. (1986). Platelets reduce coronary microvascular permeability to macromolecules. Am. J. Physiol..

[137] Heffner J.E., Cook J.A., Halushka P.V. (1989). Human platelets modulate edema formation in isolated rabbit lungs. J. Clin. Invest..

[138] Lo S.K., Burhop K.E., Kaplan J.E., Malik A.B. (1988). Role of platelets in maintenance of pulmonary vascular permeability to protein. Am. J. Physiol..

[139] Aursnes I. (1974). Increased permeability of capillaries to protein during thrombocytopenia. An experimental study in the rabbit. Microvasc. Res..

[140] Gupta S., Konradt C., Corken A., Ware J., Nieswandt B., Di Paola J., Yu M., Wang D., Nieman M.T., Whiteheart S.W., Brass L.F. (2020). Hemostasis vs. homeostasis: platelets are essential for preserving vascular barrier function in the absence of injury or inflammation. Proc. Natl. Acad. Sci. U.S.A..

[141] Nachman R.L., Rafii S. (2008). Platelets, petechiae, and preservation of the vascular wall. New England J. Med..

[142] Gros A., Ollivier V., Ho-Tin-Noé B. (2014). Platelets in inflammation: regulation of leukocyte activities and vascular repair. Front. Immunol..

[143] Petri B., Broermann A., Li H., Khandoga A.G., Zarbock A., Krombach F., Goerge T., Schneider S.W., Jones C., Nieswandt B., Wild M.K., Vestweber D. (2010). von Willebrand factor promotes leukocyte extravasation. Blood.

[144] Hidalgo A., Chang J., Jang J.E., Peired A.J., Chiang E.Y., Frenette P.S. (2009). Heterotypic interactions enabled by polarized neutrophil microdomains mediate thromboinflammatory injury. Nat. Med..

[145] Cloutier N., Paré A., Farndale R.W., Schumacher H.R., Nigrovic P.A., Lacroix S. (2012). Platelets can enhance vascular permeability. Blood.

[146] Stokes K.Y., Granger D.N. (2012). Platelets: a critical link between inflammation and microvascular dysfunction. J. Physiol..

[147] Margraf A., Zarbock A. (2019). Platelets in inflammation and resolution. J. Immunol..

[148] Baimukanova G., Miyazawa B., Potter D.R., Muench M.O., Bruhn R., Gibb S.L. (2016). Platelets regulate vascular endothelial stability: assessing the storage lesion and donor variability of apheresis platelets. Transfusion..

[149] Baimukanova G., Miyazawa B., Potter D.R., Gibb S.L., Keating S., Danesh A. (2016). The effects of 22°C and 4°C storage of platelets on vascular endothelial integrity and function. Transfusion..

[150] Devine D.V., Serrano K. (2010). The platelet storage lesion. Clin. Lab. Med..

[151] Miyazawa B., Trivedi A., Togarrati P.P., Potter D., Baimukanova G., Vivona L., Lin M., Lopez E., Callcut R., Srivastava A.K., Kornblith L.Z., Fields A.T., Schreiber M.A., Wade C.E., Holcomb J.B., Pati S. (2019). Regulation of endothelial cell permeability by platelet-derived extracellular vesicles. J. Trauma Acute Care Surg.

[152] Trivedi A., Potter D.R., Miyazawa B.Y., Lin M., Vivona L.R., Khakoo M.A. (2020). Freeze dried platelets promote clot formation, rescue thrombin induced endothelial cell permeability, and decrease pulmonary vascular leak in a murine model of hemorrhagic shock. J. Trauma Acute Care Surg..

[153] Potter D.R., Damiano E.R. (2008). The hydrodynamically relevant endothelial cell glycocalyx observed in vivo is absent in vitro. Circ. Res..

[154] Holcomb J.B., del Junco D.J., Fox E.E., Wade C.E., Cohen M.J., Schreiber M.A., Alarcon L.H., Bai Y.u., Brasel K.J., Bulger E.M., Cotton B.A., Matijevic N., Muskat P., Myers J.G., Phelan H.A., White C.E., Zhang J., Rahbar M.H., PROMMTT Study Group F.T. (2013). The prospective, observational, multicenter, major trauma transfusion (PROMMTT) study: comparative effectiveness of a time-varying treatment with competing risks. JAMA Surg..

[155] Moore H.B., Moore E.E., Chapman M.P., McVaney K., Bryskiewicz G., Blechar R., Chin T., Burlew C.C., Pieracci F., West F.B., Fleming C.D., Ghasabyan A., Chandler J., Silliman C.C., Banerjee A., Sauaia A. (2018). Plasma-first resuscitation to treat haemorrhagic shock during emergency ground transportation in an urban area: a randomised trial. Lancet.

[156] Pusateri A.E., Moore E.E., Moore H.B., Le T.D., Guyette F.X., Chapman M.P. (2020). Association of prehospital plasma transfusion with survival in trauma patients with hemorrhagic shock when transport times are longer than 20 minutes: a post hoc analysis of the PAMPer and COMBAT Clinical Trials. JAMA Surg..

[157] Shackelford S.A., del Junco D.J., Powell-Dunford N., Mazuchowski E.L., Howard J.T., Kotwal R.S., Gurney J., Butler F.K., Gross K., Stockinger Z.T. (2017). Association of prehospital blood product transfusion during medical evacuation of combat casualties in Afghanistan With Acute and 30-Day Survival. JAMA.

[158] Watson J.J., Pati S., Schreiber M.A. (2016). Plasma transfusion: history, current realities, and novel improvements. Shock..

